# Non-motorized Treadmill Running Is Associated with Higher Cardiometabolic Demands Compared with Overground and Motorized Treadmill Running

**DOI:** 10.3389/fphys.2017.00914

**Published:** 2017-11-14

**Authors:** Robert B. Edwards, Paul J. Tofari, Stuart J. Cormack, Douglas G. Whyte

**Affiliations:** School of Exercise Science, Australian Catholic University, Melbourne, VIC, Australia

**Keywords:** running, running economy, VO_2_, oxygen consumption, treadmill, perceived exertion, lower body power, lower body strength

## Abstract

The aim of this study was to compare the cardiometabolic demands of running on a curved non-motorized treadmill (cNMT) with overground (OVR) and motorized treadmill (MOT) running. Fourteen trained male (*n* = 7) and female (*n* = 7) runners (${\overset{\cdot }{\text{V}}\text{O}}_{2\text{peak}}$ 56.6 ± 4.0 mL.kg^−1^.min^−1^) participated in the study. Each experimental session consisted of 5 × 6-min bouts of running at progressively higher speeds, separated by 6-min rest (females 9–15 km.h^−1^; males 10.5–16.5 km.h^−1^). Oxygen consumption (${\overset{\cdot }{\text{V}}\text{O}}_{2}$) and heart rate (HR) during the last 2 min of each bout were measured using a portable metabolic cart. Running at a set speed on the cNMT required a higher percentage of ${\overset{\cdot }{\text{V}}\text{O}}_{2\text{peak}}$ than OVR (mean ± 90% CI, 22 ± 6%; ES ± 90% CI, 1.87 ± 0.15) and MOT (16 ± 6%; ES 1.50 ± 0.15) running. Similarly, HR during the cNMT was higher compared to OVR (25 ± 9 beats.min^−1^, ES 1.23 ± 0.14) and MOT (22 ± 9 beats.min^−1^, ES 1.35 ± 0.13) trials. The decline in running economy observed during the cNMT trial was negatively related to body mass (*R*^2^ 0.493, *P* = 0.01), indicating lighter runners were required to work at a higher relative intensity to overcome treadmill belt resistance. These data demonstrate the higher cardiometabolic demand associated with running at a given speed on the cNMT. It is critical these differences are taken into account when prescribing training intensities on the cNMT or translating data from the laboratory to an athletic setting.

## Introduction

Non-motorized treadmills (NMTs) are becoming increasingly popular as a tool for training, clinical rehabilitation, and laboratory based research. Compared to a motorized treadmill (MOT), where belt speed is controlled by an external motor, NMTs are participant driven and provide a closer experience to overground locomotion by allowing rapid acceleration and deceleration, step-to-step gait variability and internal pacing (De Witt et al., 2009; Fullenkamp et al., 2015; Stevens et al., 2015). A number of recent studies have shown NMTs to be a practical, valid, and reliable tool for assessing a range of clinical (Janaudis-Ferreira et al., 2010) and sport-specific movement patterns; including sprinting (Gonzalez et al., 2013; Mangine et al., 2014), endurance (Davies et al., 1984; Stevens et al., 2014, 2015; Morgan et al., 2016; Waldman et al., 2017) and team-sport running (Sirotic and Coutts, 2008; Aldous et al., 2014; Tofari et al., 2015). Consequently, the last few years has seen a marked increase in the use of NMTs in laboratory based interventions investigating the impact of environmental factors (Aldous et al., 2016; Gerrett et al., 2016; Sweeting et al., 2017; Stevens et al., 2017a,b), warm up (van den Tillaar et al., 2017), recovery (Pelka et al., 2017), fatigue (Tofari et al., 2017), and ergogenic aids (Sear et al., 2010; Coull et al., 2015) on performance. However, it is still unclear how the cardiometabolic demands of running on a non- NMT compare with that of running either overground or on a MOT.

Unlike flat-belt NMTs, the Woodway Curve™ (Woodway Inc., Waukesha, WI) uses a curved belt. The concave belt design provides a number of advantages in that it does not require a harness, permitting the runner unrestricted movement, and allows runners to accelerate and decelerate using similar techniques to overground running. However, performance outcomes on the curved non-motorized treadmill (cNMT) are markedly reduced compared to those obtained either overground or on a MOT (Stevens et al., 2014; Smoliga et al., 2015; Morgan et al., 2016). For example, 5-km time-trial performance, in moderately trained runners, was shown to decrease ~20% (272 s) when performed on a cNMT compared to an outdoor running track (Stevens et al., 2014). Similarly, the peak velocity obtained during an incremental exercise test on a cNMT was ~2 km.h^−1^ slower to that achieved on a MOT (Morgan et al., 2016). In both studies the performances elicited similar cardiometabolic loads, suggesting that running on a cNMT generates a greater physiological stress for a given velocity, but the extent of this increase has yet to be fully determined.

While previous work has demonstrated that a 1% incline on a MOT best replicates the physiological demands of outdoor running (Jones and Doust, 1996), only one study has examined the relative demands of running on a cNMT. Smoliga et al. (2015) compared the physiological demands of walking (4.8 km.h^−1^) and running (8.1 km.h^−1^) on a cNMT to those of a MOT. Locomotion at either speed on the cNMT resulted in significantly higher blood lactate levels, heart rate (HR), and oxygen consumption (${\overset{\cdot }{\text{V}}\text{O}}_{2}$). While of potential relevance to clinical populations, the study's relevance to athletic populations is more limited due to the relatively slow speeds selected (Boey et al., 2017), the highest of which reflects the break point between walking and running (Falls and Humphrey, 1975), and the lack of comparison with overground locomotion.

The elevated cardiometabolic demand associated with running on the cNMT is likely due to the higher resistance of the treadmill belt and the need to accelerate the belt between each step. The force required to maintain a constant speed on a NMT increases with the runners mass, although the increase in the resistance: body mass is disproportionate, leaving lighter runners at a relative disadvantage (Lakomy, 1987). Understanding the relative differences in cardiometabolic demands of running on a cNMT compared with those of either overground or MOT running is important for athletic trainers and sports scientists in order to allow the appropriate prescription of training intensities, as well as interpretation and transfer of data obtained using a cNMT from the laboratory to the athletic environment.

Therefore, the primary aim of this study was to compare the cardiometabolic demands of running on a cNMT, across a range of athletically relevant running speeds, with those experienced either overground or on a MOT. The secondary aims of the study were to (i) assess the reliability of cardiometabolic measures obtained on a cNMT and, (ii) given the anticipated increase in belt resistance on the cNMT, determine whether any changes in ${\overset{\cdot }{\text{V}}\text{O}}_{2}$ were related to differences in lower body power or maximal strength.

## Materials and methods

### Subjects

Twenty-one (12 male and 9 female) runners, aged between 18 and 45 years and capable of running 5 km in <20 min volunteered to participate in the study. Seven runners failed to complete the required sessions or were excluded from the study due to unrelated injuries (*n* = 4), perceived breathing difficulties associated with using the metabolic cart (*n* = 2) or racing commitments (*n* = 1). Consequently the data presented in the study represent 14 subjects (7 males and 7 females). Participant characteristics are outlined in Table 1. Following the recommendations of De Pauw et al. (2013) and Decroix et al. (2016), male and female subjects were classified as performance level 3 and 4, respectively. This study was carried out in accordance with the recommendations of the Australian Government, National Health and Medical Research Council with written informed consent from all subjects. All subjects gave written informed consent in accordance with the Declaration of Helsinki. The protocol was approved by the Australian Catholic University Human Research Ethics Committee (2015-214H).

**Table 1:** Descriptive and strength characteristics of participants (*n* = 14).

	**Male *n* = 7**	**Female *n* = 7**	**Combined *n* = 14**	**ES ± 90% CI**
Age (years)	34.6 ± 6.7	28.4 ± 6.8	31.5 ± 7.2	0.70 ± 0.75
Height (cm)	178 ± 10.7	162 ± 8.8	170 ± 12.6	1.54 ± 0.89
Mass (kg)	72.1 ± 10.5	53.7 ± 6.5	62.9 ± 12.7	2.02 ± 0.91
${\overset{\cdot }{\text{V}}\text{O}}_{2}$ peak (mL.min^−1^)	4,170 ± 556	2,945 ± 282	3,558 ± 764	3.10 ± 0.99
${\overset{\cdot }{\text{V}}\text{O}}_{2}$ peak (mL.kg^−1^.min^−1^)	58.0 ± 3.7	55.1 ± 4.0	56.6 ± 4.0	0.63 ± 0.79
HR max (beats.min^−1^)	191 ± 11	180 ± 10	185 ± 12	0.87 ± 0.82
Peak Treadmill Speed (km.h^−1^)	20.6 ± 0.8	18.4 ± 1.0	19.5 ± 1.4	2.49 ± 0.89
IMTP peak (N.kg^−1^)	37.4 ± 4.9	29.6 ± 3.6	33.5 ± 5.8	1.70 ± 0.89
CMJ peak power (W.kg^−1^)	44.7 ± 5.6	34.6 ± 4.7	39.6 ± 7.2	1.67 ± 0.82
SJ peak power (W.kg^−1^)	43.5 ± 6.7	33.5 ± 4.8	38.5 ± 7.7	1.57 ± 0.84

*Data presented are means ± SD for all variables. Effect size (ES) ± 90% confidence intervals (CI) are presented for the difference between males and females participants*.

### Procedures

**Figure 1:**
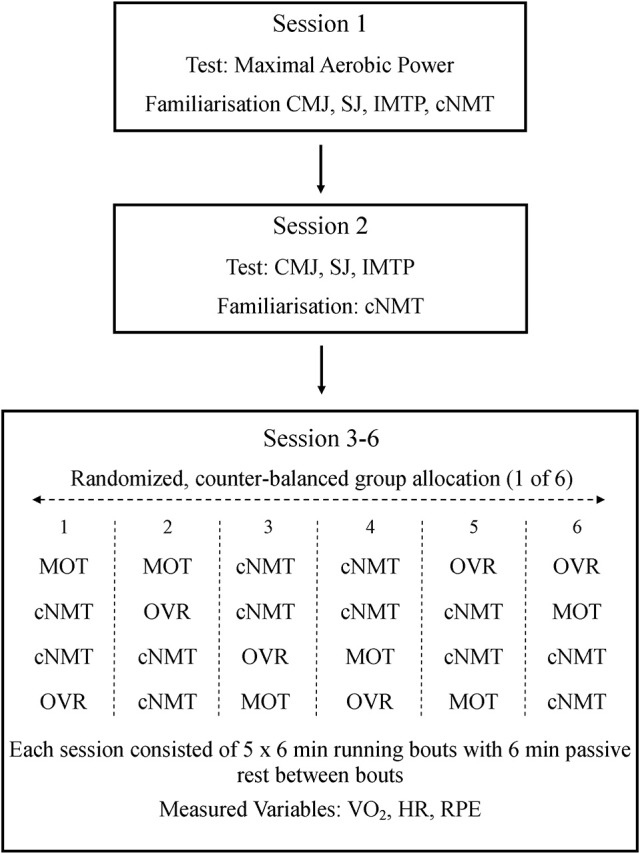
Study design. Metabolic data were monitored throughout each of the running bouts and RPE collected at the end of each bout. CMJ, counter movement jump; cNMT, curved non-motorized treadmill; HR, heart rate; IMTP, isometric mid-thigh pull; MOT, motorized treadmill; OVR, overground; RPE, rating of perceived exertion; SJ, squat jump; VO_2_, oxygen consumption.

Each subject completed six experimental sessions. Subjects arrived at the laboratory for each session in a fasted state, having abstained from caffeine for 12 h, and alcohol and strenuous exercise for 24 h prior to each session. In the first session, maximal aerobic power (VO_2peak_) was determined and subjects were familiarized with the strength testing protocols and running on the cNMT. In the second session, subjects performed the strength tests and completed a second familiarization on the cNMT to ensure data reliability (Gonzalez et al., 2013; Tofari et al., 2015). Familiarization trials on the cNMT consisted of running for 2 min at each of the required speeds. Each running bout was separated by 1 min of passive rest except for the final bout, which was preceded by 4 min of passive rest. The final four sessions comprised the experimental trials; overground (OVR), motorized treadmill (MOT), and cNMT running. Trials were completed in a randomized, counter-balanced manner, at the same time of day and with at least 48 h between experimental sessions to ensure adequate recovery. Two cNMT trials were completed in order to assess the reliability of the physiological measures and the subjects' ability to maintain the appropriate treadmill speed. The two cNMT trials were completed on consecutive sessions within the randomized trial order (Figure 1).

An incremental exercise test, performed on a MOT (HP cosmos, Nussdorf-Traunstein, Germany) set at a 1% gradient, was used to determine ${\overset{\cdot }{\text{V}}\text{O}}_{2\text{peak}}$. After 3 min at either 10 km.h^−1^ (male) or 8 km.h^−1^ (female) treadmill speed was increased by 1 km.h^−1^ every minute until volitional exhaustion. Female subjects started the incremental exercise test at a slower initial speed in order to maintain similar test durations between groups (Schabort et al., 2000). Expired air was measured breath by breath using a portable metabolic cart (K4b^2^, Cosmed, Rome, Italy) and the data averaged over 30 s periods (Robergs et al., 2010). Heart rate was monitored continuously (FT1, Polar, Finland) throughout each trial and subjects rated their perceived level of exertion (RPE) in the final 15 s of each stage using the Borg 6–20 scale (Borg, 1982). The highest average VO_2_ recorded over 30 s in either the incremental exercise test or steady state trials was defined as the subjects ${\overset{\cdot }{\text{V}}\text{O}}_{2\text{peak}}$ and used for subsequent calculations.

Countermovement (CMJ) and squat jumps (SJ) and isometric mid-thigh pull (IMTP) performance were assessed to determine if lower body power or maximal strength influenced the degree of change in VO_2_ observed between the running trials. All tests were performed in triplicate using a force platform (400 series, Fitness Technology, Adelaide, Australia) and a sampling rate of 600 Hz (Ballistic Measurement System v 2015.0.0, Fitness Technology, Adelaide, Australia). Prior to each testing session the force platform was leveled and a two-point calibration performed. Subjects performed three CMJ from a standing position, with their hands on their hips throughout the movement. During SJ, subjects were instructed to squat with their hands on their hips and wait (~3 s pause) for the command “jump,” before jumping for maximum height. The SJ trial was repeated if a counter movement >5% body mass was detected. Each jump trial was separated by 1 min of passive rest and the trial with the greatest peak power used for data analysis. A 6 s IMTP was performed using a mid-thigh pull rig (Fitness Technology, Adelaide, Australia) and the trial with the highest peak force used for analysis. Each IMTP trial was separated by 3 min passive rest.

In each of the experimental trials, male (10.5, 12, 13.5, 15, 16.5 km.h^−1^) and female (9, 10.5, 12, 13.5, 15 km.h^−1^) subjects completed 5 × 6-min runs, in ascending order, separated by 6 min of passive rest (Jones and Doust, 1996). The five speeds represented running velocities of 50 ± 2, 58 ± 2, 66 ± 3, 73 ± 3, and 81 ± 3% of subjects peak treadmill velocity. Ventilatory variables (K4b^2^, Cosmed, Rome, Italy) and HR were collected continuously throughout each 6-min trial and data from the final 2 min used for analysis. Artifactual breaths were filtered prior to analysis using a two stage process. Respiratory frequencies 3.5 times greater (140 breaths.min^−1^) than reported maximal respiratory rates (Blackie et al., 1991) were initially excluded before the data were filtered using a threshold set at 3 *SD* from the mean ${\overset{\cdot }{\text{V}}\text{O}}_{2}$ (Lamarra et al., 1987). Subjects indicated their RPE at the conclusion of each stage. Running economy was compared between trials conducted at 10.5 km.h^−1^ using data from the final 2 min of the stage. This speed was selected as it was common between both males and females and there were 12 subjects who completed the cNMT trial at this speed with an RER < 1.0. Running economy was expressed as oxygen unit cost (mL.kg^−1^.km^−1^).

Overground trials were completed on a wooden floored, indoor sports stadium to minimize environmental influences on performance. A 144 m track was set out using a 14 m radius to create two 43.98 m curved ends and 28.02 m straights, preventing sharp directional changes. Timing lights (Smartspeed, Fusion Sport, Sumner Park, Australia) were placed every 12 m around the track for visual pacing and a single timing gate on the start/finish line was used to collect lap splits for analysis of running speed. The treadmill (Pulsar, HP Cosmos, Nussdorf-Traunstein, Germany) used in the MOT trials was set at a 1% gradient (Jones and Doust, 1996). Accuracy of the belt speed was checked using a video camera and found to be within 0.03–0.07 m.s^−1^ (<1.5%) of the prescribed speed. Pacing during the cNMT (Curve 3, Woodway, Waukesha, WI) trials was maintained using a visual pacer (Pacer performance system, Innervations, Australia) projected onto a large screen in front of the treadmill. In order to maintain the correct running speed, subjects matched a pacing line, representing their current speed, to a line indicating the required speed. This same software allowed the collection of the cNMT belt speed, at a sample rate of 200 Hz, for reliability assessment.

### Statistical analyses

All data are presented as mean ± standard deviation. An initial sample size of 12 was estimated based on an *a priori* power test (G Power, v 3.0.10) using previously published data (Smoliga et al., 2015) and designed to achieve an α = 0.005 and β = 0.90. A contemporary analytical approach involving magnitude-based inferences was used to detect important effects between the different trials (Batterham and Hopkins, 2006). Using a customized spreadsheet (Hopkins, 2003), data were log-transformed to account for non-uniformity of error and differences between trials assessed using the effect size (ES) statistic, with 90% confidence intervals using the combined standard deviations of male and female groups. The magnitude of difference between the means was classified as practically “*important*” when there was ≥75% likelihood that the true value of the statistic exceeded a threshold ES-value (0.2) (Batterham and Hopkins, 2006). Differences with less certainty were classified as “*trivial*,” and when the likelihood of the statistic occurring simultaneously in both directions was >5%, the effect was reported as “*unclear*” (Batterham and Hopkins, 2006). Subsequently, a multiple regression analysis (forward method) was used to determine if; (i) body mass; and (ii) measures of relative strength and power contributed to the change in %${\overset{\cdot }{\text{V}}\text{O}}_{2\text{peak}}$ during running on the cNMT (IBM SPSS Statistics v22; SPSS Inc., Chicago, IL, USA). The inter-trial reliability of the mean cNMT belt, physiological and perceptual variables at each of the speed increments was estimated by calculating the typical error and expressing it as a percentage [coefficient of variation (CV%)] ± 90% confidence limits (CL) (Hopkins, 2000).

## Results

Descriptive characteristics of the participants are shown in Table 1. Male runners were older, taller and had greater body mass than the female runners. The absolute ${\overset{\cdot }{\text{V}}\text{O}}_{2\text{peak}}$, peak treadmill velocity, and measures of relative lower body power and strength were also higher in males. However, the difference in relative ${\overset{\cdot }{\text{V}}\text{O}}_{2\text{peak}}$ between males and females was *unclear*.

All subjects successfully completed each of the five required speeds in the OVR and MOT trials. However, only one participant (male) completed the entire cNMT trial, and only 6 of the 14 runners could maintain the penultimate speed for 6 min on the cNMT (4 males, 2 females). Consequently, statistical comparisons involving the cNMT only include treadmill speeds between 9 and 13.5 km.h^−1^, whereas comparisons between the MOT and OVR trials use data from all speeds.

### Reliability

In order to compare between the different running modalities it was critical that participants reliably maintained the appropriate speed and were at steady state when physiological data were collected. Set running speeds were maintained within 0.02 and 0.01 m.s^−1^ on the cNMT and OVR trials, respectively. Belt speed between the two cNMT trials was reliable (CV 0.19–0.51%) and within <1% of each of the target speeds (Table 2). The range of CV% for physiological variables between cNMT trials was 1.36–3.03% (Table 2). Perceived exertion was the least reliable (CV 2.06–7.71%), although this represents a difference in RPE score of ~1 unit (Table 2). Comparison of ${\overset{\cdot }{\text{V}}\text{O}}_{2}$ and HR data between minutes five and six across all speeds revealed only *trivial* differences during all of the trials and near perfect relationships (Table 3). Therefore, subjects were deemed to be at steady state and data from the final 2 min were combined for further analysis.

**Table 2:** Reliability of physiological, perceptual, and performance variables between cNMT trials.

	**Male**			**Female**			**Overall**		
	***n***	**% CV**	**90% CL**	***n***	**% CV**	**90% CL**	***n***	**% CV**	**90% CL**
**ABSOLUTE VO**_2_									
9.0 km.h^−1^	−	−	−	6	3.03	2.03−6.43	−	−	−
10.5 km.h^−1^	7	2.61	1.80−5.07	6	1.39	0.93−2.93	13	1.93	1.46−2.94
12.0 km.h^−1^	7	2.78	1.91−5.39	5	2.52	1.63−6.07	12	2.66	1.98−4.15
13.5 km.h^−1^	7	2.97	2.04−5.76	−	−	−	8	2.74	1.93−4.99
15.0 km.h^−1^	4	1.98	1.22−5.90	−	−	−	−	−	−
**HEART RATE**									
9.0 km.h^−1^	−	−	−	6	2.02	1.36−4.28	−	−	−
10.5 km.h^−1^	7	2.50	1.72−4.84	6	1.88	1.26−3.96	13	2.24	1.68 −2.41
12.0 km.h^−1^	7	1.88	1.30−3.64	5	1.52	0.99−3.65	12	1.77	1.32−2.76
13.5 km.h^−1^	7	1.35	0.93−2.60	−	−	−	8	1.36	0.96−2.46
15.0 km.h^−1^	4	1.63	1.01−4.84	−	−	−	−	−	−
**RPE**									
9.0 km.h^−1^	−	−	−	6	7.65	5.08−16.64	−	−	−
10.5 km.h^−1^	7	5.63	3.85−11.06	6	7.71	5.12−16.80	13	7.23	5.42−11.17
12.0 km.h^−1^	7	6.66	4.55−13.15	5	2.06	1.33−4.96	12	5.07	3.77−7.97
13.5 km.h^−1^	7	6.25	4.28−12.32	−	−	−	8	5.93	4.15−10.91
15.0 km.h^−1^	4	5.49	3.37−16.89	−	−	−	−	−	−
**SPEED**									
9.0 km.h^−1^	−	−	−	6	0.25	0.16−0.60	−	−	−
10.5 km.h^−1^	7	0.51	0.35−0.98	6	0.19	0.13−0.40	13	0.41	0.31−0.61
12.0 km.h^−1^	7	0.30	0.21−0.57	5	0.35	0.32−0.83	12	0.32	0.24−0.50
13.5 km.h^−1^	7	0.25	0.17−0.47	−	−	−	8	0.25	0.17−0.47
15.0 km.h^−1^	4	0.27	0.16−0.78	−	−	−	−	−	−

**Table 3:** Mean oxygen consumption (${\overset{\cdot }{\text{V}}\text{O}}_{2}$) and heart rate (HR) during the last 2 min of all trials during cNMT, MOT and OVR running.

	**OVR**			**MOT**			**cNMT**		
	**5th min**	**6th min**	***r***	**5th min**	**6th min**	***r***	**5th min**	**6th min**	***r***
VO_2_ (L.min^−1^)	2.62 ± 0.78	2.64 ± 0.79	0.997	2.78 ± 0.75	2.80 ± 0.76	0.997	3.18 ± 0.71	3.20 ± 0.73	0.999
HR (beats.min^−1^)	151 ± 22	152 ± 23	0.998	155 ± 22	156 ± 22	0.990	164 ± 17	166 ± 17	0.994

### Physiological responses

**Figure 2:**
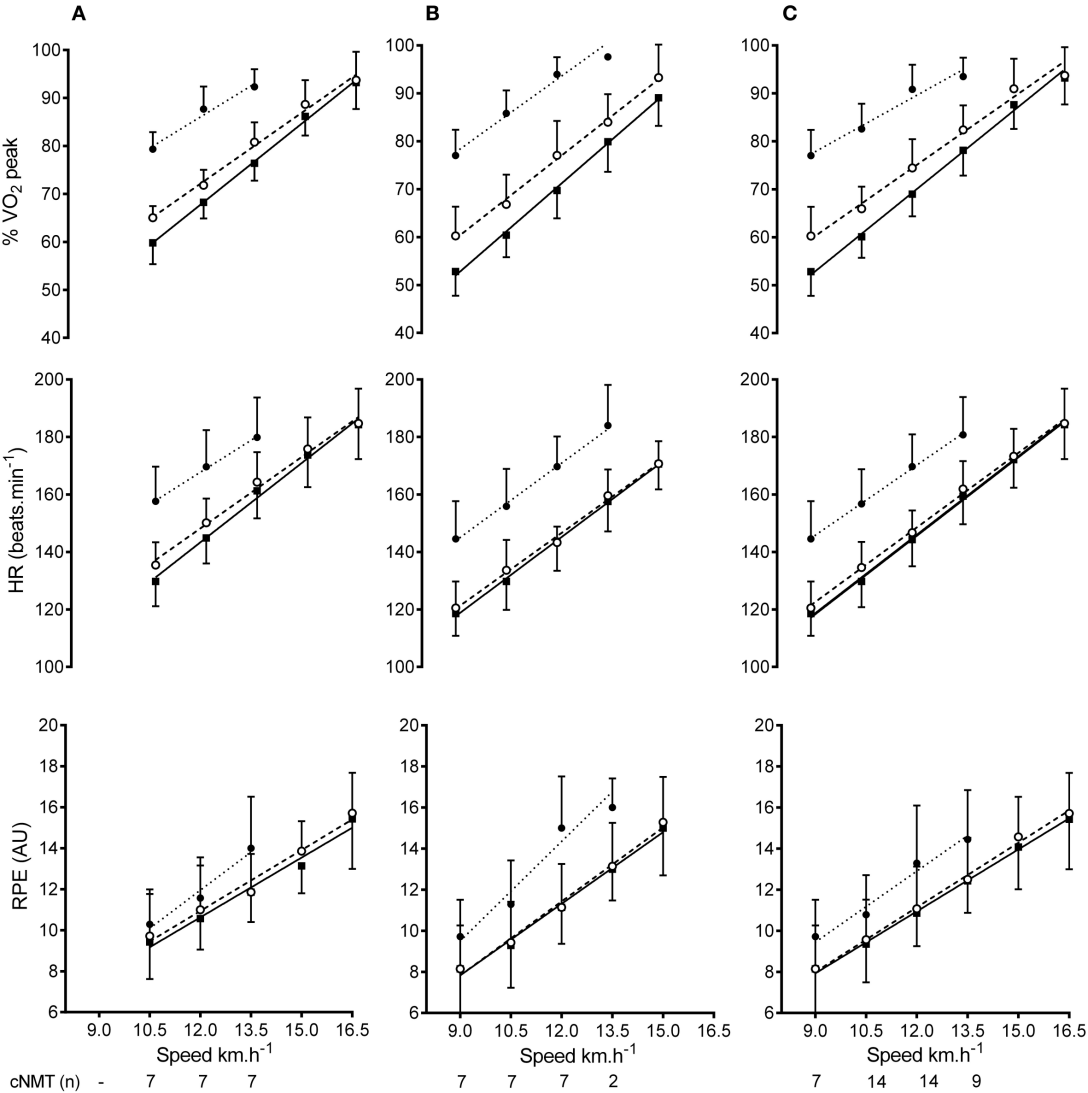
Male **(A)**, female **(B)**, and combined data **(C)** for percent peak oxygen consumption (%VO_2peak_), heart rate (HR) and rating of perceived exertion (RPE) at each speed during overground (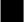) motorized (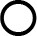) and curved non-motorized treadmill (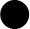) running. All data are mean ± *SD*.

Physiological and perceptual data from the different trials are presented in Figure 2 and the associated ES ± 90% CI reported in Table 4. Differences in %VO_2_, HR, and RPE between male and female runners across all speeds were either *trivial* or *unclear* in the OVR and MOT trials. However, throughout the cNMT trial males worked at a lower %VO_2_ (ES −0.70 ± 0.57) and reported a lower RPE (ES −0.51 ± 0.53) compared to females, whereas the difference in HR remained *unclear* (ES 0.22 ± 0.54). When male and female data were combined across all speeds, the average %VO_2peak_ and HR (Figure 2) were higher in the cNMT compared to the OVR (mean ± SD, %VO_2peak_ 22 ± 6%; HR 25 ± 9 beats.min^−1^) and MOT (%VO_2peak_ 16 ± 6%; HR 22 ± 9 beats.min^−1^) trials. While the average %VO_2peak_ was also higher in the MOT compared to the OVR trial (5 ± 6%), the difference in HR was *trivial* (2 ± 4 beats.min^−1^).

**Table 4:** Effect size (ES) and 90% confidence intervals (CI) of the difference in physiological and perceptual variables during cNMT, MOT, and OVR running.

	**cNMT vs. OVR**			**cNMT vs. MOT**			**MOT vs. OVR**		
	**Male ES ± CI (*n*)**	**Female ES ± CI (*n*)**	**Overall ES ± CI**	**Male ES ± CI (*n*)**	**Female ES ± CI (*n*)**	**Overall ES ± CI**	**Male ES ± CI (*n*)**	**Female ES ± CI (*n*)**	**Overall ES ± CI**
**% VO**_2peak_									
9.0 km.h^−1^	–	3.43 ± 0.55 (7)	–	–	2.12 ± 0.47 (7)	–	–	1.19 ± 0.46 (7)	–
10.5 km.h^−1^	3.36 ± 0.74 (7)	3.97 ± 0.63 (7)	4.13 ± 0.52	4.68 ± 0.85 (7)	2.31 ± 0.65 (7)	3.01 ± 0.50	1.02 ± 0.73 (7)	1.13 ± 0.59 (7)	1.21 ± 0.46
12.0 km.h^−1^	1.91 ± 0.59 (7)	2.50 ± 0.33 (7)	3.91 ± 0.51	3.86 ± 1.03 (7)	1.84 ± 0.74 (7)	2.37 ± 0.50	0.90 ± 0.85 (7)	1.04 ± 0.65 (7)	1.07 ± 0.52
13.5 km.h^−1^	3.54 ± 0.73 (7)	3.28 ± 1.84 (2)	3.03 ± 0.60	2.26 ± 0.64 (7)	2.23 ± 4.05^*^ (2)	2.17 ± 0.52	1.05 ± 1.09 (7)	0.55 ± 0.48 (7)	0.75 ± 0.45
15.0 km.h^−1^	1.86 ± 1.18 (4)	–	1.64 ± 1.04	0.95 ± 0.67 (4)	–	0.86 ± 0.60	0.53 ± 0.91^*^ (7)	0.61 ± 0.69 (7)	0.61 ± 0.52
16.5 km.h^−1^	–	–	–	–	–	–	0.07 ± 1.08^*^ (7)	–	–
Combined	1.99 ± 0.24 (21)	1.71 ± 0.18 (21)	1.87 ± 0.15	1.70 ± 0.23 (21)	1.33 ± 0.19 (21)	1.50 ± 0.15	0.26 ± 0.13 (35)	0.40 ± 0.10 (35)	0.33 ± 0.08
**HEART RATE**									
9.0 km.h^−1^	–	2.67 ± 0.59 (7)	2.67 ± 0.59	–	2.03 ± 0.63 (6)	2.03 ± 0.63	–	0.10 ± 0.20^†^ (6)	–
10.5 km.h^−1^	2.58 ± 0.68 (7)	2.15 ± 0.75 (7)	2.64 ± 0.50	2.24 ± 0.54 (7)	1.79 ± 0.81 (6)	2.26 ± 0.48	0.58 ± 0.36 (7)	0.29 ± 0.23 (6)	0.49 ± 0.22
12.0 km.h^−1^	2.25 ± 0.56 (7)	2.06 ± 0.58 (7)	2.41 ± 0.40	1.91 ± 0.39 (7)	3.70 ± 1.06 (7)	2.64 ± 0.48	0.49 ± 0.28 (7)	−0.01 ± 0.39^*^ (7)	0.25 ± 0.28
13.5 km.h^−1^	1.57 ± 0.33 (7)	1.84 ± 0.94 (2)	1.77 ± 0.29	1.27 ± 0.28 (7)	1.83 ± 1.85 (2)	1.56 ± 0.28	0.26 ± 0.17 (7)	0.20 ± 0.26 (7)	0.25 ± 0.15
15.0 km.h^−1^	0.86 ± 0.17 (4)	–	1.05 ± 0.21	0.66 ± 0.10 (4)	–	0.81 ± 0.12	0.18 ± 0.15 (7)	0.02 ± 0.23^*^ (7)	0.12 ± 0.14^†^
16.5 km.h^−1^	–	–	–	–	–	–	0.02 ± 0.15^†^ (7)	–	–
Combined	1.36 ± 0.21(21)	1.37 ± 0.18 (21)	1.23 ± 0.14	1.19 ± 0.16 (21)	1.34 ± 0.21 (19)	1.35 ± 0.13	0.15 ± 0.05^†^ (35)	0.06 ± 0.05^†^ (33)	0.10 ± 0.04^†^
**RPE**									
9.0 km.h^−1^	–	0.63 ± 0.50 (7)	0.63 ± 0.50	–	0.65 ± 0.30 (7)	0.65 ± 0.30	–	0.01 ± 0.41^*^ (7)	–
10.5 km.h^−1^	0.38 ± 0.32 (7)	0.78 ± 0.44 (7)	0.66 ± 0.30	0.25 ± 0.31 (7)	0.73 ± 0.46 (7)	0.55 ± 0.30	0.11 ± 0.30^†^ (7)	0.07 ± 0.13^†^ (7)	0.10 ± 0.15^†^
12.0 km.h^−1^	0.51 ± 0.59 (7)	1.58 ± 0.42 (7)	1.19 ± 0.47	0.22 ± 0.33 (7)	1.31 ± 0.45 (7)	0.83 ± 0.39	0.17 ± 0.70^*^ (7)	−0.03 ± 0.23^*^ (7)	0.07 ± 0.35^*^
13.5 km.h^−1^	1.02 ± 0.61 (7)	2.46 ± 0.90 (2)	1.42 ± 0.60	0.79 ± 0.23 (7)	1.95 ± 0.93 (2)	1.13 ± 0.37	−0.04 ± 0.37^*^ (7)	0.04 ± 0.56^*^ (7)	0.00 ± 0.30^*^
15.0 km.h^−1^	1.44 ± 0.30 (4)	–	1.15 ± 0.24	1.06 ± 0.48 (4)	–	0.95 ± 0.43	0.33 ± 0.22 (7)	0.11 ± 0.27^†^ (7)	0.24 ± 0.17
16.5 km.h^−1^	–	–	–	–	–	–	0.12 ± 0.40^*^ (7)	–	–
Combined	0.58 ± 0.24 (21)	0.86 ± 0.20 (21)	0.74 ± 0.16	0.42 ± 0.18 (21)	0.84 ± 0.18 (21)	0.67 ± 0.14	0.11 ± 0.12 (35)	0.00 ± 0.09^†^ (35)	0.06 ± 0.07^†^

Combined data is 9.0–13.5 km.h^−1^ for cNMT comparisons and 9.0–16.5 km.h^−1^ for MOT and OVR. All data reflect an important difference unless otherwise identified as either a Trivial, or ^*^unclear difference.

### Perceptual responses

The perceived intensity of running on the cNMT was higher compared to the OVR (2 ± 2 AU) and MOT (2 ± 2 AU) trials, whereas the difference between RPE during OVR and MOT was *trivial* (0 ± 1). Consequently, the %VO_2_: RPE was higher in the cNMT (7.18 ± 1.46; ES 0.64 ± 0.28) and MOT (6.83 ± 1.45; ES 0.36 ± 0.21) compared to OVR (6.44 ± 0.87) trials, but only a *trivial* difference existed between the cNMT and MOT (ES 0.10 ± 0.23) trials. Differences in the HR: RPE across all trials were either *trivial* or *unclear*.

### Running economy

**Figure 3:**
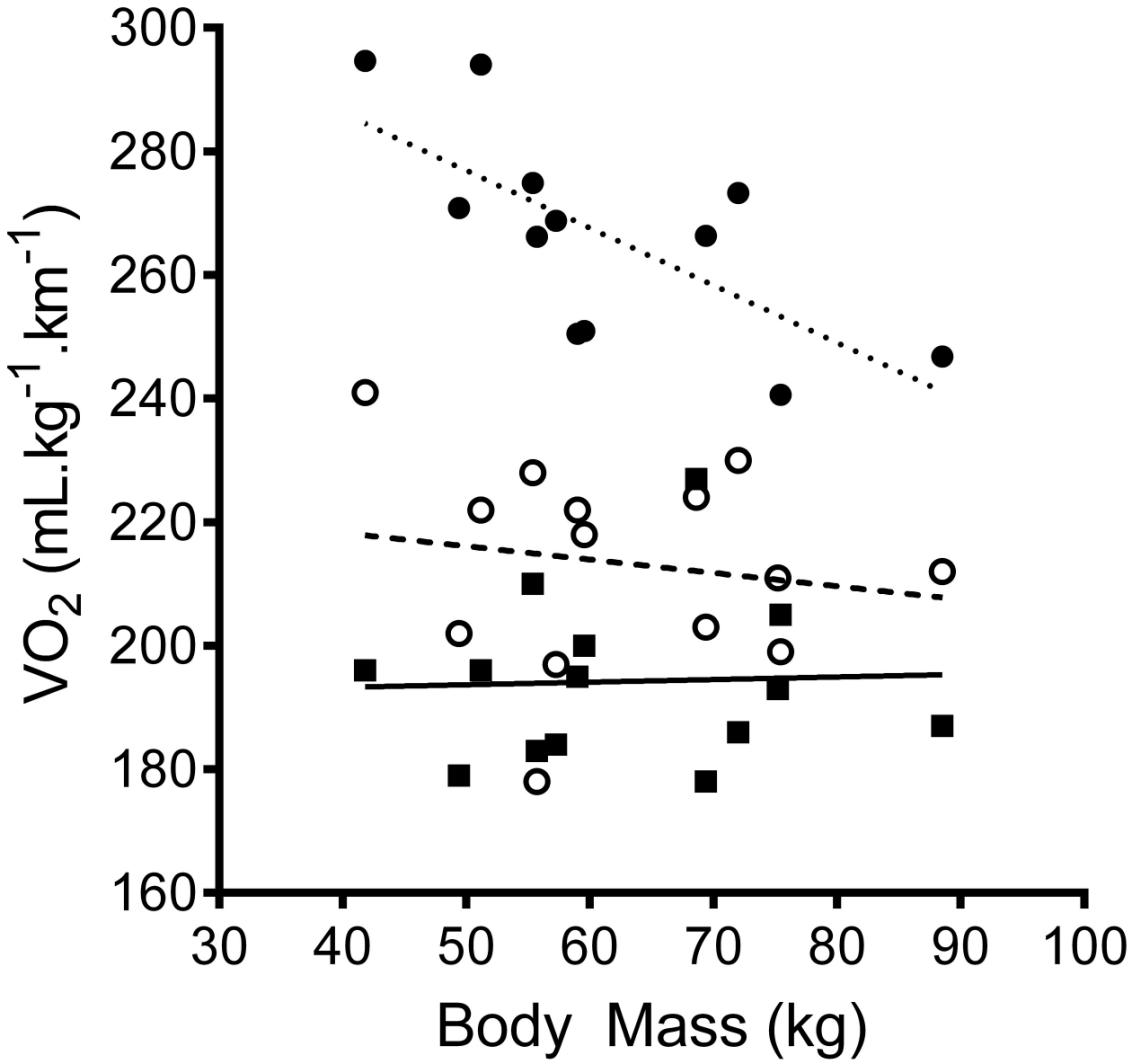
The relationship between running economy and body mass at 10.5 km.h^−1^ during overground (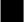, solid line) motorized (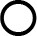, dashed line) and curved non-motorized treadmill (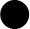, dotted line) running.

Differences in running economy between males and females were *unclear* in the OVR (ES −0.50 ± 0.91), MOT (ES −0.25 ± 0.90), and cNMT (ES 0.61 ± 0.98). Overall, running economy during the OVR (194 ± 13 mL.kg^−1^.km^−1^) was markedly better when compared to MOT (213 ± 16 mL.kg^−1^.km^−1^; ES 1.31 ± 0.67) and cNMT (266 ± 17 mL.kg^−1^.km^−1^; ES 4.45 ± 0.62). Similarly, economy during the MOT trial was better when compared to cNMT (ES 2.70 ± 0.57). A significant negative relationship between body mass and running economy was found in the cNMT trials (VO_2_ = −0.93^*^mass + 323.56; *r* = −0.70, *P* = 0.01). This relationship was not present in either the OVR (VO_2_ = 0.04^*^mass + 191.57; *r* = 0.039, *P* = 0.89) or MOT (VO_2_ = −0.22^*^mass + 226.89; *r* = −0.17, *P* = 0.57) trials (Figure 3) and was not improved by measures of lower body power and strength.

## Discussion

The purpose of the current study was to compare the cardiometabolic and perceptual responses to running on a cNMT with those observed during MOT and OVR running. The results demonstrate that, when matched for speed, running on the cNMT generates a much larger cardiometabolic stress than either MOT or OVR in both male and female runners. The decrease in running economy was negatively related to body mass, indicating lighter runners found running on the cNMT more demanding than heavier runners. Relative lower body strength and power did not appear to influence the degree of change in running economy.

Both ${\overset{\cdot }{\text{V}}\text{O}}_{2}$ and HR increased linearly with running speed in all trials and were markedly higher in the cNMT trial. A number of previous studies have alluded to an increase in the cardiometabolic demand associated with walking (Seneli et al., 2013; Smoliga et al., 2015) and running (Stevens et al., 2014; Smoliga et al., 2015; Morgan et al., 2016) on a cNMT compared to either a MOT or OVR. This increase in demand was starkly demonstrated in the current study by the inability of all but one of the runners to complete the required intervals on the cNMT, despite all participants completing the identical speeds in the OVR and MOT trials. While the majority of participants were unable to complete the cNMT trial, the average ${\overset{\cdot }{\text{V}}\text{O}}_{2}$ during the last completed stage equated to 96 ± 3% of their ${\overset{\cdot }{\text{V}}\text{O}}_{2\text{peak}}$, and was marginally higher than the levels attained during either the MOT (93 ± 6%) and OVR (91 ± 6%) trials. In addition, RPE were similar at the end of the final completed stage in all trials (OVR 15.2 ±2.3; MOT 15.5 ±2.0; cNMT 15.8 ±1.8) indicating a high degree of effort.

Only one other study has directly examined the physiological and perceptual demands of locomotion on a cNMT. Smoliga et al. (2015) compared walking (4.8 km.h^−1^) and running (8.1 km.h^−1^) on a cNMT to a MOT and reported an increase in absolute ${\overset{\cdot }{\text{V}}\text{O}}_{2}$ (0.6 and 0.8 L.min^−1^) and HR (21 and 31 beats.min^−1^) at both speeds. The increase in ${\overset{\cdot }{\text{V}}\text{O}}_{2}$ (0.5 L. min^−1^) and HR (25 beats.min^−1^) observed in the current study when comparing running at the slowest speed (9 km.h^−1^) on the cNMT to the MOT were slightly lower than those reported by Smoliga et al. (2015). Across all speeds the increase in oxygen consumption equated to an increase in the relative level of oxygen consumption of ~15 % VO_2peak_. This discrepancy is likely because we used a 1% gradient during the MOT trial, increasing the relative demand compared to the 0% gradient used by Smoliga et al. (2015) and thereby reducing the difference between the cNMT and MOT trials.

The largest difference in ${\overset{\cdot }{\text{V}}\text{O}}_{2}$ and HR were observed between the cNMT and OVR trials and equated to an increase in the relative level of oxygen consumption of ~20% of VO_2peak_. The larger decrease in running economy observed during the cNMT trial is likely due to the need to overcome the inertial load of the cNMT belt. The horizontal force needed to maintain a constant speed on a NMT increases with runner mass (Lakomy, 1987). However, the increase in resistance is not directly proportional to body mass, leaving lighter runners at a disadvantage as they need to produce a greater relative increase in force and power to overcome the belt resistance at any given speed (Lakomy, 1987). The very strong negative relationship between participant body mass and the decrease in running economy observed in the current study (Figure 3) suggests a similar relationship exists on the cNMT and is in agreement with previous work showing a positive relationship between body mass and running performance on the cNMT (Stevens et al., 2014). Indeed, the higher ${\overset{\cdot }{\text{V}}\text{O}}_{2}$ observed in the female runners, compared to the males, during the cNMT trial is likely a reflection of their lighter body mass (~18 kg, Table 1). Based on the increase in absolute ${\overset{\cdot }{\text{V}}\text{O}}_{2}$ at 10.5 km.h^−1^ during the MOT (10%) and cNMT (37%) trials, the average running speed would need to be decreased by 1.1 and 4.1 km.h^−1^, respectively, in order to maintain the same relative intensity as that during OVR (Figure 2) (Burkett et al., 1985). These estimated decreases in running speed are larger than those observed during a 5 km time trial performed on a cNMT, where runners decreased their speed by ~2.5 km.h^−1^ in order to maintain a similar internal load (Stevens et al., 2014), but may simply be due to the lower average body mass in our subjects.

The addition of measures of lower body power and maximal strength did not provide any further explanation of the change in running economy above that of body mass itself, suggesting that at least within this population, differences in relative strength and power were not of sufficient magnitude to overcome any additional increase in belt resistance. However, it should be noted that the current study was not appropriately powered to rule out a role for these variables, and was also limited by a relatively homogenous subject pool. While the performance related inclusion criteria were necessary to ensure participants could complete a number of stages on the cNMT future studies should consider targeting a more diverse group of athletes, particularly those with larger body mass and relative strength, such as team-sport athletes.

In addition to the inherent resistance of the belt, runners on a cNMT manipulate belt speed by landing at different points on the curve. For example, to accelerate the belt runners move closer to the front, initially landing on an area of the belt angled at 5–10° above horizontal. Oxygen consumption increases with grade on a MOT (Jones and Doust, 1996) and it has been suggested the incline of the cNMT belt may also contribute to the increased intensity (Smoliga et al., 2015); however, the degree to which this variable contributes likely varies both between and within subjects, as stride length and frequency is varied in order to maintain the correct speed, making its contribution difficult to determine.

Decreases in running economy were also seen when MOT was compared with OVR. This finding supports those of earlier studies which suggest the decrease in economy during treadmill running is due to less economic movement patterns and the subsequent increase in ventilatory work (Meyer et al., 2003; Mooses et al., 2015). However, the degree of difference between the two trials may also have been exaggerated by equipment related factors. For example, in the MOT trial treadmill gradient was set at a 1% to compensate for the lack of wind resistance (Jones and Doust, 1996). While this level of gradient is well-accepted in the literature, treadmill belt compliance also makes an important contribution to running economy and was not taken into consideration in the current study (Smith et al., 2017).

Not surprisingly, given the higher cardiometabolic demand, participants perceived the cNMT trials to be harder than the MOT and OVR. These data are consistent with previous studies that have shown self-selected running speeds on a cNMT are slower than those on either a MOT (Smoliga et al., 2015) or OVR (Stevens et al., 2014) as subjects adjust their speed to achieve a similar internal load. However, our findings are in direct contrast to those of Morgan et al. (2016) who found no difference in RPE during 75% of the stages of an incremental exercise test performed on either a MOT or cNMT, despite significant differences in ${\overset{\cdot }{\text{V}}\text{O}}_{2}$ and HR. Females perceived running on the cNMT to be harder than males at all speeds. This was likely related to the higher %VO_2peak_ required due to their lower body mass, as when RPE was expressed relative to %VO_2peak_ there was no difference between males and females. The %VO_2peak_: RPE was, however, higher in both the MOT and cNMT trials when compared to OVR. Thus, participants perceived a higher metabolic demand as slightly easier in either of the treadmill conditions. This shift in the perceived intensity of a given metabolic load is difficult to explain but is likely due to a conflict between sensory inputs and an individual's prior experience. Treadmill running does not provide the runner with the usual optic flow, thus depriving the subjects of an important source of pacing feedback (Pelah and Barlow, 1996). This lack of a visual representation of speed, combined with the relative novelty of treadmill running and the related potential changes in running kinetics and kinematics, may have led to an altered judgement of the exercise intensity (Kong et al., 2012).

Irrespective of the cause of the increase in physiological strain, the data from this study clearly demonstrate running speeds derived from exercise tests performed on MOT or OVR need to be carefully considered before being used to prescribe exercise programs on a cNMT. Direct transposition of absolute speeds derived from OVR or MOT running performance may result in the prescription of training loads that induce a far greater physiological strain than intended, increasing the risk of injury and overtraining and likely make sessions difficult or potentially impossible to complete. Basing training intensity on RPE is also potentially problematic, as while perceived effort was higher during the cNMT, the relationship between ${\overset{\cdot }{\text{V}}\text{O}}_{2}$ and RPE was also altered. Thus, prescriptions based on RPE may result in athletes working at exercise intensities that require on average 9% more of their VO_2peak_ to accomplish on the cNMT. This relative reduction in perceived effort, which has been noted previously during steady state and maximal exercise tests using a cNMT (Smoliga et al., 2015; Morgan et al., 2016), could also prove advantageous when trying to increase the metabolic load of a training program without the concomitant increase in effort.

A secondary aim of the study was to examine the reliability of cardiometabolic data collected when running at different speeds on the cNMT. Participants completed two familiarization sessions to ensure they could maintain the correct pacing and run confidently on the cNMT without the need for handrail support (Sirotic and Coutts, 2008; Mangine et al., 2014; Tofari et al., 2015). Using the visual pacer, subjects were able to reliably maintain cNMT belt speed within 0.02 m.s^−1^ of the target speed, a similar accuracy to that previously reported for walking and jogging on a cNMT (Smoliga et al., 2015). The measured physiological variables were also highly reliable between tests (CV%: ${\overset{\cdot }{\text{V}}\text{O}}_{2}$ 1.4–3.0, HR 1.4–2.5), similar to those previously reported in submaximal MOT running [CV%: ${\overset{\cdot }{\text{V}}\text{O}}_{2}$ 2.4–2.5, HR 1.7–2.4 (Saunders et al., 2004)] and cNMT based time trials [CV%: ${\overset{\cdot }{\text{V}}\text{O}}_{2}$ 2.7–4.3, HR 1.1–2.1 (Stevens et al., 2015)] and well above the previously reported levels of reliability for the Cosmed system itself (Duffield et al., 2004). Thus, this study demonstrates that reliable performance and cardiometabolic data can be obtained during visually paced running across a range of speeds on a cNMT after two familiarization sessions.

### Limitations and perspectives

By only including subjects capable of running 5 km in <20 min, we recruited a relatively homogeneous pool of endurance runners, with a comparatively low body mass. Given the relationship between body mass and the relative force required to overcome belt resistance, this may have inflated the difference in exercise intensity observed. Therefore, the degree to which ${\overset{\cdot }{\text{V}}\text{O}}_{2}$ increases when running on a cNMT may be lower in individuals with greater mass, such as male team-sport athletes. Furthermore, the performance related inclusion criteria restricted the size of the participant pool and the low participant numbers meant the study was inadequately powered to fully investigate any potential relationships between performance variables (e.g., lower body power, strength) and the change in ${\overset{\cdot }{\text{V}}\text{O}}_{2}$.

## Conclusion

Non-motorized treadmills provide an attractive alternative to training on a MOT as they allow a closer approximation of overground running in terms of pacing and gait. However, the results of the current study demonstrate that at any given submaximal speed, running on a cNMT provides a markedly higher cardiometabolic stress compared to running on either a MOT or overground. This is particularly true for female athletes whose lower body mass may put them at a disadvantage in overcoming the treadmill belt resistance. Therefore, when prescribing exercise on a cNMT, it is critical that relationship between running speed and exercise intensity, as well as the athletes body mass are considered in order generate an appropriate internal load and training stimulus.

## Author contributions

Conception and design of experiments: RE, PT, SC, and DW. Performance of experiments: RE, PT, and DW. Data analysis: RE and DW. Preparation and approval of final manuscript: RE, PT, SC, and DW.

### Conflict of interest statement

The authors declare that the research was conducted in the absence of any commercial or financial relationships that could be construed as a potential conflict of interest.

## Abbreviations

CMJ: counter movement jump
cNMT: curved non-motorized treadmill
IMTP: isometric mid-thigh pull
MOT: motorized treadmill
OVR: overground
SJ: squat jump.

## References

[B1] Aldous J. W. Akubat I. Chrismas B. C. Watkins S. L. Mauger A. R. Midgley A. W.. (2014). The reliability and validity of a soccer-specific nonmotorised treadmill simulation intermittent soccer performance test. J. Strength Cond. Res. 28, 1971–1980. 10.1519/JSC.0000000000000310 24169475

[B2] Aldous J. W. Chrismas B. C. Akubat I. Dascombe B. Abt G. Taylor L. (2016). Hot and hypoxic environments inhibit simulated soccer performance and exacerbate performance decrements when combined. Front. Physiol. 6:421. 10.3389/fphys.2015.00421 26793122 PMC4709924

[B3] Batterham A. M. Hopkins W. G. (2006). Making meaningful inferences about magnitudes. Int. J. Sports Physiol. Perf. 1, 50–57. 10.1123/ijspp.1.1.50 19114737

[B4] Blackie S. P. Fairbarn M. S. McElvaney N. G. Wilcox P. G. Morrison N. J. Pardy R. L. (1991). Normal values and ranges for ventilation and breathing pattern at maximal exercise. Chest 100, 136–142. 10.1378/chest.100.1.136 1905613

[B5] Boey H. Aeles J. Schütte K. Vanwanseele B. (2017). The effect of three surface conditions, speed and running experience on vertical acceleration of the tibia during running. Sports Biomech. 16, 166–176. 10.1080/14763141.2016.1212918 27595311

[B6] Borg G. A. (1982). Psychophysical bases of perceived exertion. Med. Sci. Sports Exerc. 14, 377–381. 10.1249/00005768-198205000-00012 7154893

[B7] Burkett L. Kohrt W. M. Buchbinder R. (1985). Effects of shoes and foot orthotics on VO_2_ and selected frontal plane knee kinematics. Med. Sci. Sports Exerc. 17, 158–163. 10.1249/00005768-198502000-00026 3982270

[B8] Coull N. A. Watkins S. L. Aldous J. W. Warren L. K. Chrismas B. C. Dascombe B.. (2015). Effect of tyrosine ingestion on cognitive and physical performance utilising an intermittent soccer performance test (iSPT) in a warm environment. Eur. J. Appl. Physiol. 115, 373–386. 10.1007/s00421-014-3022-7 25326727

[B9] Davies B. Daggett A. Jakeman P. Mulhall J. (1984). Maximum oxygen uptake utilising different treadmill protocols. Br. J. Sports Med. 18, 74–79. 10.1136/bjsm.18.2.74 6466933 PMC1859190

[B10] De Pauw K. Roelands B. Cheung S. S. de Geus B. Rietjens G. Meeusen R. (2013). Guidelines to classify subject groups in sport-science research. Int. J. Sports Physiol. Perf. 8, 111–122. 10.1123/ijspp.8.2.111 23428482

[B11] De Witt J. K. Lee S. M. Wilson C. A. Hagan R. D. (2009). Determinants of time to fatigue during nonmotorized treadmill exercise. J. Strength Cond. Res. 23, 883–890. 10.1519/JSC.0b013e3181a04de9 19387389

[B12] Decroix L. De Pauw K. Foster C. Meeusen R. (2016). Guidelines to classify female subject groups in sport-science research. Int. J. Sports Physiol. Perf. 11, 204–213. 10.1123/ijspp.2015-0153 26182438

[B13] Duffield R. Dawson B. Pinnington H. Wong P. (2004). Accuracy and reliability of a cosmed K4b 2 portable gas analysis system. J. Sci. Med. Sport 7, 11–22. 10.1016/S1440-2440(04)80039-2 15139160

[B14] Falls H. B. Humphrey L. D. (1975). Energy cost of running and walking in young women. Med. Sci. Sports 8, 9–13. 1272008

[B15] Fullenkamp A. M. Matthew Laurent C. Campbell B. M. (2015). Automated gait temporal-spatial assessment from non-motorized treadmill belt speed data. Gait Posture 41, 141–145. 10.1016/j.gaitpost.2014.09.017 25311386

[B16] Gerrett N. Jackson S. Yates J. Thomas G. (2016). Ice slurry ingestion does not enhance self-paced intermittent exercise in the heat. Scand. J. Med. Sci. Sports 27, 1202–1212. 10.1111/sms.12744 27624363

[B17] Gonzalez A. M. Wells A. J. Hoffman J. R. Stout J. R. Fragala M. S. Mangine G. T.. (2013). Reliability of the Woodway curve non-motorized treadmill for assessing anaerobic performance. J. Sports Sci. Med. 12, 104–108. 24149732 PMC3761752

[B18] Hopkins W. (2003). A spreadsheet for analysis of straightforward controlled trials. Sports Sci. 7:15.

[B19] Hopkins W. G. (2000). Measures of Reliability in Sports Medicine and Science 30, 1–15. 10.2165/00007256-200030010-00001 10907753

[B20] Janaudis-Ferreira T. Sundelin G. Wadell K. (2010). Comparison of the 6-minute walk distance test performed on a non-motorised treadmill and in a corridor in healthy elderly subjects. Physiotherapy 96, 234–239. 10.1016/j.physio.2009.11.015 20674656

[B21] Jones A. M. Doust J. H. (1996). A 1% treadmill grade most accurately reflects the energetic cost of outdoor running. J. Sports Sci. 14, 321–327. 10.1080/02640419608727717 8887211

[B22] Kong P. W. Koh T. M. Tan W. C. Wang Y. S. (2012). Unmatched perception of speed when running overground and on a treadmill. Gait Posture 36, 46–48. 10.1016/j.gaitpost.2012.01.001 22357398

[B23] Lakomy H. (1987). The use of a non-motorized treadmill for analysing sprint performance. Ergonomics 30, 627–637. 10.1080/00140138708969756

[B24] Lamarra N. Whipp B. J. Ward S. A. Wasserman K. (1987). Effect of interbreath fluctuations on characterizing exercise gas exchange kinetics. J. Appl. Physiol. 62, 2003–2012. 3110126 10.1152/jappl.1987.62.5.2003

[B25] Mangine G. T. Hoffman J. R. Gonzalez A. M. Wells A. J. Townsend J. R. Jajtner A. R.. (2014). Speed, force, and power values produced from nonmotorized treadmill test are related to sprinting performance. J. Strength Cond. Res. 28, 1812–1819. 10.1519/JSC.0000000000000316 24950225

[B26] Meyer T. Welter J.-P. Scharhag J. Kindermann W. (2003). Maximal oxygen uptake during field running does not exceed that measured during treadmill exercise. Eur. J. Appl. Physiol. 88, 387–389. 10.1007/s00421-002-0718-x 12527967

[B27] Mooses M. Tippi B. Mooses K. Durussel J. Mäestu J. (2015). Better economy in field running than on the treadmill: evidence from high-level distance runners. Biol. Sport 32, 155–159. 10.5604/20831862.1144418 26060340 PMC4447762

[B28] Morgan A. L. Laurent M. Fullenkamp A. M. (2016). Comparison of VO2 peak performance on a motorized versus a non-motorized treadmill. J. Strength Cond. Res. 30, 1898–1905. 10.1519/JSC.0000000000001273 27328274

[B29] Pelah A. Barlow H. (1996). Visual illusion from running. Nature 381, 283. 10.1038/381283a0 8692265

[B30] Pelka M. Kölling S. Ferrauti A. Meyer T. Pfeiffer M. Kellmann M. (2017). Acute effects of psychological relaxation techniques between two physical tasks. J. Sports Sci. 35, 216–223. 10.1080/02640414.2016.1161208 26999625

[B31] Robergs R. A. Dwyer D. Astorino T. (2010). Recommendations for improved data processing from expired gas analysis indirect calorimetry. Sports Med. 40, 95–111. 10.2165/11319670-000000000-00000 20092364

[B32] Saunders P. U. Pyne D. B. Telford R. D. Hawley J. A. (2004). Reliability and variability of running economy in elite distance runners. Med. Sci. Sport Exerc. 36, 1972–1976. 10.1249/01.MSS.0000145468.17329.9F 15514515

[B33] Schabort E. J. Killian S. C. St Clair Gibson A. Hawley J. A. Noakes T. D. (2000). Prediction of triathlon race time from laboratory testing in national triathletes. Med. Sci. Sports Exerc. 32, 844–849. 10.1097/00005768-200004000-00018 10776905

[B34] Sear J. A. Hoare T. K. Scanlan A. T. Abt G. A. Dascombe B. J. (2010). The effects of whole-body compression garments on prolonged high-intensity intermittent exercise. J. Strength Cond. Res. 24, 1901–1910. 10.1519/JSC.0b013e3181db251b 20555284

[B35] Seneli R. M. Ebersole K. O'Connor K. Snyder A. C. (2013). Estimated VO_2_max from the rockport walk test on a non-motorized curved treadmill. J. Strength Cond. Res. 27, 3495–3505. 10.1519/JSC.0b013e31828f04d8 23478472

[B36] Sirotic A. C. Coutts A. J. (2008). The reliability of physiological and performance measures during simulated team-sport running on a non-motorised treadmill. J. Sci. Med. Sport 11, 500–509. 10.1016/j.jsams.2007.04.008 17706459

[B37] Smith J. A. H. McKerrow A. D. Kohn T. A. (2017). Metabolic cost of running is greater on a treadmill with a stiffer running platform. J. Sports Sci. 35, 1592–1597. 10.1080/02640414.2016.1225974 27575734

[B38] Smoliga J. M. Hegedus E. J. Ford K. R. (2015). Increased physiologic intensity during walking and running on a non-motorized, curved treadmill. Phys. Ther. Sport 16, 262–267. 10.1016/j.ptsp.2014.09.001 25824428

[B39] Stevens C. J. Bennett K. J. Sculley D. V. Callister R. Taylor L. Dascombe B. J. (2017a). A comparison of mixed-method cooling interventions on preloaded running performance in the heat. J. Strength Cond. Res. 31, 620–629. 10.1519/JSC.0000000000001532 27379961

[B40] Stevens C. J. Hacene J. Wellham B. Sculley D. V. Callister R. Taylor L. (2014). The validity of endurance running performance on the Curve 3^TM^ non-motorised treadmill. J. Sports Sci. 33, 1141–1148. 10.1080/02640414.2014.986502 25490348

[B41] Stevens C. J. Kittel A. Sculley D. V. Callister R. Taylor L. Dascombe B. J. (2017b). Running performance in the heat is improved by similar magnitude with pre-exercise cold-water immersion and mid-exercise facial water spray. J. Sports Sci. 35, 798–805. 10.1080/02640414.2016.1192294 27267974

[B42] Stevens C. J. Hacene J. Sculley D. V. Taylor L. Callister R. Dascombe B. (2015). The reliability of running performance in a 5 km time trial on a non-motorized treadmill. Int. J. Sports Med. 36, 705–709. 10.1055/s-0034-1398680 25790087

[B43] Sweeting A. J. Billaut F. Varley M. C. Rodriguez R. F. Hopkins W. G. Aughey R. J. (2017). Variations in hypoxia impairs muscle oxygenation and performance during simulated team-sport running. Front. Physiol. 8:80. 10.3389/fphys.2017.00080 28239359 PMC5301029

[B44] Tofari P. J. McLean B. D. Kemp J. Cormack S. (2015). A self-paced intermittent protocol on a non-motorised treadmill: a reliable alternative to assessing team-sport running performance. J. Sports Sci. Med. 14, 62–68. 25729291 PMC4306784

[B45] Tofari P. Kemp J. Cormack S. (2017). A self-paced team sport match simulation results in reductions in voluntary activation and modifications to biological, perceptual and performance measures at half-time, and for up to 96 hours post-match. J. Strength Cond. Res. [Epub ahead of print]. 10.1519/JSC.0000000000001875 28240711

[B46] van den Tillaar R. Vatten T. von Heimburg E. (2017). Effects of short or long warm-up on intermediate running performance. J. Strength Cond. Res. 31, 37–44. 10.1519/JSC.0000000000001489 27191697

[B47] Waldman H. S. Heatherly A. J. Waddell A. F. Krings B. M. O'Neal E. K. (2017). 5-km Time trial reliability of a non-motorized treadmill and comparison of physiological and perceptual responses versus a motorized treadmill. J. Strength Cond. Res. [Epub ahead of print]. 10.1519/JSC.0000000000001993 28542090

